# Denitrification Biokinetics: Towards Optimization for Industrial Applications

**DOI:** 10.3389/fmicb.2021.610389

**Published:** 2021-05-05

**Authors:** Navreet Suri, Yuan Zhang, Lisa M. Gieg, M. Cathryn Ryan

**Affiliations:** ^1^Department of Geoscience, University of Calgary, Calgary, AB, Canada; ^2^Department of Biological Sciences, University of Calgary, Calgary, AB, Canada

**Keywords:** denitrification, *Thauera*, NO_3_^–^ concentration, NO_2_^–^ accumulation, pH, denitrification gene transcripts, souring, MEOR

## Abstract

Denitrification is a microbial process that converts nitrate (NO_3_^–^) to N_2_ and can play an important role in industrial applications such as souring control and microbially enhanced oil recovery (MEOR). The effectiveness of using NO_3_^–^ in souring control depends on the partial reduction of NO_3_^–^ to nitrite (NO_2_^–^) and/or N_2_O while in MEOR complete reduction of NO_3_^–^ to N_2_ is desired. *Thauera* has been reported as a dominant taxon in such applications, but the impact of NO_3_^–^ and NO_2_^–^ concentrations, and pH on the kinetics of denitrification by this bacterium is not known. With the goal of better understanding the effects of such parameters on applications such as souring and MEOR, three strains of *Thauera* (K172, NS1 and TK001) were used to study denitrification kinetics when using acetate as an electron donor. At low initial NO_3_^–^ concentrations (∼1 mmol L^–1^) and at pH 7.5, complete NO_3_^–^ reduction by all strains was indicated by non-detectable NO_3_^–^ concentrations and near-complete recovery (> 97%) of the initial NO_3_-N as N_2_ after 14 days of incubation. The relative rate of denitrification by NS1 was low, 0.071 mmol L^–1^ d^–1^, compared to that of K172 (0.431 mmol L^–1^ d^–1^) and TK001 (0.429 mmol L^–1^ d^–1^). Transient accumulation of up to 0.74 mmol L^–1^ NO_2_^–^ was observed in cultures of NS1 only. Increased initial NO_3_^–^ concentrations resulted in the accumulation of elevated concentrations of NO_2_^–^ and N_2_O, particularly in incubations with K172 and NS1. Strain TK001 had the most extensive NO_3_^–^ reduction under high initial NO_3_^–^ concentrations, but still had only ∼78% of the initial NO_3_-N recovered as N_2_ after 90 days of incubation. As denitrification proceeded, increased pH substantially reduced denitrification rates when values exceeded ∼ 9. The rate and extent of NO_3_^–^ reduction were also affected by NO_2_^–^ accumulation, particularly in incubations with K172, where up to more than a 2-fold rate decrease was observed. The decrease in rate was associated with decreased transcript abundances of denitrification genes (*nirS* and *nosZ*) required to produce enzymes for reduction of NO_2_^–^ and N_2_O. Conversely, high pH also contributed to the delayed expression of these gene transcripts rather than their abundances in strains NS1 and TK001. Increased NO_2_^–^ concentrations, N_2_O levels and high pH appeared to cause higher stress on NS1 than on K172 and TK001 for N_2_ production. Collectively, these results indicate that increased pH can alter the kinetics of denitrification by *Thauera* strains used in this study, suggesting that liming could be a way to achieve partial denitrification to promote NO_2_^–^ and N_2_O production (e.g., for souring control) while pH buffering would be desirable for achieving complete denitrification to N_2_ (e.g., for gas-mediated MEOR).

## Introduction

Denitrification is a major nitrate (NO_3_^–^) reduction pathway that contributes to biochemical gas (NO, N_2_O and N_2_) production in anoxic environments. It is a microbially facilitated process performed by facultative anaerobic bacteria capable of reducing NO_3_^–^ to nitrite (NO_2_^–^), ultimately producing di-nitrogen (N_2_) via other gas species (NO and N_2_O; Korom, 1992). The complete reduction of NO_3_^–^ to N_2_ requires the presence of sufficient amounts of organic (for organoheterotrophic denitrification; like organic acids, hydrocarbons) and/or inorganic (for lithotrophic denitrification; like sulfur containing compounds, transition metals) electron donors (Spormann and Widdel, 2000; Chakraborty and Coates, 2004; Zhu and Getting, 2012; Capua et al., 2019). The transient accumulation of intermediates (NO_2_^–^, NO, and N_2_O) can be observed at the onset of or during denitrification, under optimum growth and metabolic conditions. On the other hand, accumulated NO_2_^–^ and N_2_O can persist at sub-optimal and extreme environmental conditions (e.g., high temperatures, acidic or alkaline pH conditions, high salinities; Liu et al., 2014; Fida et al., 2016; An et al., 2017). Limited availability of electron donors can also drive partial denitrification, but many natural denitrifier habitats contain rich sources of electron donors.

Denitrification is widespread in nature and the production of NO_2_^–^, N_2_O and/or N_2_ as end products is required in many NO_3_^–^-dependent practical uses. This metabolic process can play an important role in controlling sulfide formation and microbially enhanced oil recovery (MEOR) in oil reservoirs. Souring control can be achieved by NO_2_^–^ inhibition of dissimilatory sulfite reductase (Dsr) required for the reduction of sulfite to sulfide by sulfate-reducing bacteria (SRB; Gieg et al., 2011; Callbeck et al., 2013). Nitrous oxide (N_2_O) in its dissolved form can also be beneficial to limiting sulfide production since it is toxic to SRB and many other microbial species by raising the redox potential in its ambient environment (Sørensen et al., 1980; Londry and Suflita, 1999). However, the liberation of excess N_2_O to the atmosphere is a concern because of its high potency for ozone depletion in the stratosphere and contribution to greenhouse gas levels (Ravishankara et al., 2009; Montzka et al., 2011). Nitrite can also cause toxicity to other organisms. Thus, the complete reduction of produced NO_2_^–^ and N_2_O to N_2_ is preferred in many industrial applications, including NO_3_^–^ removal from contaminated waters and gas-mediated enhanced oil recovery (Soares, 2000; Rocca et al., 2007; Lazar et al., 2007; Safdel et al., 2017; Huno et al., 2018).

The production of denitrification intermediates and N_2_ is dependent on physico-chemical growth conditions, enzyme kinetics and differential gene expression patterns among denitrifying species (Philippot et al., 2001; Liu et al., 2013; Fida et al., 2016). The majority of denitrifying bacteria are distributed within the classes α-, β-, γ-, and ε- of phylum *Proteobacteria* though they can be members of other phyla such as *Firmicutes*, *Actinobacteria*, *Bacteroidetes* and *Planctomyces* (Zumft, 1997). Several genera of these bacteria have been enriched and isolated from different environments to determine rates of denitrification and their abilities to express denitrification genes. Their phenotypic responses vary more with diverse regulatory networks than the genes controlling denitrification outcomes (Zumft, 1997; Van Spanning et al., 2007). Under mesophilic conditions, members of the genus *Thauera* within the *Betaproteobacteria* have been reported to be dominant in NO_3_^–^-impacted environments (Agrawal et al., 2012; Shen et al., 2018). Some strains of this genus were isolated and characterized for denitrification products and their denitrification regulatory phenotypes (DRPs; Liu et al., 2013; Fida et al., 2016). *Thauera* can use volatile fatty acids (VFAs such as acetate, propionate and butyrate), monoterpenes, heterocyclic aromatic compounds and hydrocarbons as electron donors for organoheterotrophic NO_3_^–^ reduction (Foss and Harder, 1998; Mechichi et al., 2002). Two distinct types of DRPs have been distinguished in *Thauera*, based on the accumulation of NO_2_^–^ as an intermediate when NO_3_^–^ reduction occurs with acetate oxidation. These include rapid, complete onset (RCO) or progressive onset (PO) of denitrification genes. The regulatory control of N_2_O was observed to be less stringent than the other intermediates among all the *Thauera* strains irrespective of their DRPs. Although the N_2_O intermediate remained as a major end product in the incubations of one of these strains (*T. phenylacetica*; Liu et al., 2013), it was not clear whether this difference was related to its phylogeny, specific DRP, or physico-chemical growth conditions. This particular study (Liu et al., 2013) did not include strains like *T. aromatica* that are often the dominant denitrifiers in different environments, and which have been found to be important mediators of NO_3_^–^-dependent sulfide control and MEOR (Evans et al., 1991; Anders et al., 1995; Mechichi et al., 2002; Agrawal et al., 2012; Suri et al., 2019).

To further extend the knowledge on regulatory phenotypes of other *Thauera* strains and the factors affecting NO_3_^–^ reduction to N_2_ production in the interest of application to industrial processes, *Thauera* strains isolated from organic carbon-rich environments were selected for the current study. The known optimum growth temperature and pH range for these strains is 28–30°C and 7.0–7.5, respectively. Incomplete to complete reduction of 1 to 5 mmol L^–1^ NO_3_^–^ using VFAs, esters and alkylbenzenes as electron donors by these strains was shown in previous studies under these optimal conditions (Mechichi et al., 2002; Fida et al., 2016; Suri et al., 2017). The partial reduction of NO_3_^–^ to NO_2_^–^, and prolonged NO_2_^–^ accumulation was observed at NO_3_^–^ concentrations higher than 2 mmol L^–1^, but the role of N_2_O and N_2_ cannot be assessed since their concentrations were not reported. In addition, the effect of important growth parameters (e.g., pH) on their metabolic abilities to produce N_2_ was not addressed (Fida et al., 2016; Suri et al., 2017; Suri et al., 2019). The variable production of NO_2_^–^, N_2_O and N_2_ by denitrifiers is desired in different industrial applications, which can potentially be customized by modification of their growth environments. Keeping the published data in view, the impact of initial NO_3_^–^ concentrations, intermediate denitrification products (NO_2_^–^, N_2_O), and culture pH values on the rate and extent of denitrification were evaluated for the growth of representative *Thauera* strains. In addition, NO_2_^–^ toxicity to the *Thauera* stains was evaluated by using it as an electron acceptor.

## Materials and Methods

### Denitrifying Bacterial Strains

Three strains of *Thauera* were used in this study (Table 1). Two of these namely, *T. aminoaromatica* TK001 and *T. aromatica* NS1, were isolated from enrichment cultures obtained using produced water from a NO_3_^–^ treated heavy oil reservoir (Fida et al., 2016; Suri et al., 2019). The third strain, *T. aromatica* K172, originated from activated sludge and was purchased from DSMZ culture collection, Braunschweig, Germany^1^.

**TABLE 1:** Description of denitrifying *Thauera* strains used to inoculate microcosms in this study.

Species	Strain	Source	Location	16S rRNA sequence depository	Accession number	References
*T. aromatica*	K172	Anaerobic sludge	Ulm, Baden-Württemberg, Germany	EMBL	X77118	Tschech and Fuchs, 1987
*T. aromatica*	NS1	Produced water	Medicine Hat, Alberta, Canada	GenBank	MK085068	Suri et al., 2019
*T. aminoaromatica*	TK001	Produced water	Medicine Hat, Alberta, Canada	GenBank	KU057961	Fida et al., 2016

### Culture Medium and Growth Conditions

All strains were grown in CSBK medium (a minimal salts medium; Suri et al., 2017) for comparison of their denitrification characteristics. After autoclaving, the medium was allowed to cool to room temperature while being flushed with 99.9% helium (He) to remove dissolved oxygen. The cooled medium was amended with pre-sterilized stock solutions of trace elements and tungstate and selenite (Widdel and Bak, 1992). Sodium bicarbonate (0.03 M) was added as a buffer and the initial pH of medium was adjusted to 7.5 by addition of 1 M hydrochloric acid (HCl).

Microcosms were prepared by dispensing 50 mL anoxic medium into 120 mL serum bottles using the Hungate technique (Löffler et al., 2005). The sterilized anaerobic stock solutions of sodium acetate (1 M) and sodium nitrate (1 M) were prepared aseptically with ultrapure (milliQ^®^) water. Microcosms were prepared using two different acetate and NO_3_^–^ concentrations (0.5 and 1 mmol L^–1^), with the same concentrations of acetate and NO_3_^–^ (1:1) added to each individual microcosm. The serum bottles were sealed with butyl rubber stoppers and crimped with aluminum seals.

Frozen bacterial stocks of *Thauera* strains were initially used for cultivation in CSBK with 0.5 mmol L^–1^ of NO_3_^–^ and acetate. Incubations were conducted at 30°C until the NO_3_^–^ and reduced intermediates were not detectable in microcosms (using the analytical methods described in sections “Biochemical Analyses” and “Headspace Gas Measurements”). The bacterial cultures were centrifuged, the collected cell pellets were re-suspended in CSBK, and were then used to inoculate duplicate or triplicate microcosms containing 1 mmol L^–1^ of NO_3_^–^ and acetate (1:1) to monitor denitrification kinetics. Prior to inoculation, the ∼70 mL headspace of each bottle was flushed with He and equilibrated to laboratory barometric pressure (∼0.9 atm) by piercing the butyl rubber stopper with a sterile syringe needle.

Bacterial cells were added to microcosms to an optical density (OD_600_) of approximately 0.005 in each bottle to attain similar initial cell densities for each of the three strains. The microcosms were then incubated at 30°C, and bacterial growth and changes in concentrations of acetate, NO_3_^–^, NO_2_^–^, N_2_O and N_2_ were monitored by withdrawing aqueous and gas phase samples periodically from the bottles during incubation using He-flushed syringes following procedures outlined in sections “Biochemical Analyses” and “Headspace Gas Measurements.”

### Biochemical Analyses

Culture sub-samples (0.5–1 mL) were collected during the incubation period to assess bacterial growth and substrate consumption rates. The aqueous samples were centrifuged in microcentrifuge tubes at 13,000 rpm for 10 min, and the collected supernatants were analyzed for acetate, NO_3_^–^, and NO_2_^–^ concentrations using High Performance Liquid Chromatography (HPLC; Waters 600E; Suri et al., 2017; Okpala and Voordouw, 2018). The remaining cell pellets were washed with sterilized distilled water twice and re-suspended in 1 mL of water. The bacterial cell concentrations were subsequently estimated by measuring optical density of cell suspensions at 600 nm (OD_600_), using distilled water as a blank.

Acetate concentrations were measured by HPLC using 300 μL of the supernatant aliquots that were acidified with 20 μL of 1 M H_3_PO_4_ prior to being eluted through a Prevail organic acid (OA) 5 μ column (250 mm × 4.6 mm, Alltech, Guelph, ON, Canada) using 25 mM KH_2_PO_4_ (pH 2.5) at a flow rate of 1 mL min^–1^. Supernatants (100 μL) mixed with 400 μL of acetonitrile buffer were eluted through an IC-PAK anion column (4 × 150 mm, Waters) with 24% (v/v) acetonitrile, 2% butanol and 2% borate-gluconate buffer at a flow rate of 2 mL min^–1^ for measuring NO_3_^–^ and NO_2_^–^. The peaks detected using a Waters 2487 UV detector at 210 and 220 nm were compared with known standards to obtain acetate, NO_3_^–^ and NO_2_^–^ concentrations, respectively.

The pH of aqueous phase samples was measured using a Thermo Scientific Inc., Orion model 310 pH meter (VWR International, Mississauga, ON, Canada) calibrated to a pH range of 4–10. Alkalinity was determined by titration of aqueous phase samples with phthalate buffer in the presence of bromophenol blue to an end point pH of 3.5. Absorbance of bromophenol blue complex measured at 600 nm (A_600_) compared to the known standards was used as a measure of total alkalinity expressed as millieq/L. An automated pre-calibrated titrator equipped with a spectrophotometer (Thermoscientific) was used for these measurements.

### Headspace Gas Measurements

Headspace gases (CO_2_, N_2_O and N_2_) were quantified using gas chromatography (GC; Hewlett Packard 5890A). Prior to sampling, the needle of an empty syringe was first pierced into rubber stoppers to release excess pressure in the headspace to barometric pressure (Supplementary Figure 1). The volume that the piston moved to was recorded as additional headspace volume. Samples (1 mL) withdrawn from the headspace using a gas tight syringe (Agilent) were injected into a GC equipped with a RT-Msieve 5A column (Restek-RT-19722; 30 m × 0.32 mm) and pulsed discharge detector-HIID mode (PDD-HIID). The injector, oven, and detector temperatures were set to 28°C, 30°C, and 33°C, respectively. Helium (> 99.99% purity; Praxair) served as the carrier and makeup gas. Standard curves generated in the range of 0.001 to 99.9% (equivalent to 0.002 to 20 mmol L^–1^) were used to calculate headspace concentrations of gases in the culture bottles. Aqueous concentrations of gases were calculated from the headspace concentrations using equation 1 and dimensionless Henry’s constants of 0.726, 0.528, and 0.014 for CO_2_, N_2_O and N_2_, respectively at 30°C. The constants were calculated using parameters provided by Sander (2015):

(1) C=aqC×gH

where C_*aq*_, C_*g*_, and H are the aqueous concentration (in mmol L^–1^), the headspace concentration (in mmol L^–1^) and dimensionless Henry’s constant, respectively. The Henry’s constants were further adjusted for the ionic strengths of the medium and culture conditions (Schumpe et al., 1982; Schumpe, 1993).

### Calculation of % Mole N-Species Remaining

In some instances, the data are presented as the % of a particular N-species remaining (e.g., NO_3_^–^, NO_2_^–^, N_2_O or N_2_). These values were estimated as follows (using the percent N_2_ produced from NO_3_^–^ as an example, where NO_3_^–^_INITIAL_, NO_3_^–^_FINAL_ and N_2_ produced are the initial NO_3_^–^ concentration, final NO_3_^–^ concentration and final N_2_ concentration, respectively, each expressed as mmol L^–1^)

(2) $$NO_{3\,I\,N\,I\,T\,I\,A\,L}^{-}recoveredasN_{2}\left(\%\right)=\frac{\left[N_{2\,P\,R\,O\,D\,U\,C\,E\,D}\right]}{\left(\left[N\,O_{3\,I\,N\,I\,T\,I\,A\,L}^{-}\right]-\left[N\,O_{3\,F\,I\,N\,A\,L}^{-}\right]\right)}*100$$

### Batch Microcosm Tests

Batch microcosms were prepared using 120 mL pre-sterilized serum bottles flushed with He, closed with butyl rubber stoppers and crimped with aluminum seals. In each case, sterilized solutions of acetate and N-species were added aseptically at different initial concentrations to anoxic CSBK medium and the pH was adjusted using 1 M HCl or 1 M NaOH. The headspaces of the bottles were subsequently flushed with He and equilibrated to barometric pressure prior to being inoculated with *Thauera* strains at initial cell densities *ca*. OD_600_ ≅ 0.005.

Four sets of iterative batch tests were prepared using the techniques above (sets 1 to 4; Table 2), with N-species from the three electron acceptors that participate in denitrification (i.e., NO_3_^–^, NO_2_^–^, or N_2_O), and in one case with varying initial pH values (7.5 to 10). In each case acetate was added as an electron donor in equimolar concentrations to the initial N-species (i.e., in 1:1 ratio). Two to four replicate microcosms were used for each of the three denitrifying strains in each set of batch microcosm tests. After preparation and inoculation, microcosms were incubated at 30°C for either 14, 30, or 90 days (depending on the experiment). Samples for initial aqueous (acetate, NO_3_^–^ and NO_2_^–^) and headspace concentrations (CO_2_, N_2_O and N_2_) were collected immediately after inoculation and for the final measurements at the end of the incubation period. Denitrification intermediates, alkalinity and pH were also measured with time and with sampling conducted during the incubation period as described above.

**TABLE 2:** Description of sets of microcosm batch tests conducted, including their purpose, the initial N-source (NO_3_^–^, NO_2_^–^ or N_2_O) and their concentrations, other initial conditions, and the figures and/or tables where the results are reported.

Set #	Purpose(s)	Initial N-source concentration(s)	Other initial conditions	Pertinent Figures/Tables
		(mmol L ^–1^)		
1	Denitrification biokinetics	NO_3_^–^	∼1	Tables 4, 5, Figures 1, 4, and Supplementary Figures 2, 3, 5
2	Effect of initial NO_3_^–^ concentration	NO_3_^–^	∼ 2, 3, 4, and 5	Table 5 and Figure 2, 4
3	Effect of initial NO_2_^–^ concentration	NO_2_^–^	∼ 1, 2, 3, 4, and 5	Figure 3 and Supplementary Figures 2, 4
4	Effect of initial N_2_O concentration (and pH)	N_2_O	∼ 0.1, 0.4, 0.7, 1.0	Figure 5
5	Effect of initial pH and expression of denitrification genes as a function of pH	NO_3_^–^	∼1, with NaHCO_3_ and Na_2_CO_3_ added at variable ratios to reach initial pH values that ranged from 7.5 to 10	Figure 6 and Supplementary Figure 6

### Effect of Initial pH on NO_3_^–^ Reduction to N_2_

**FIGURE 1:**
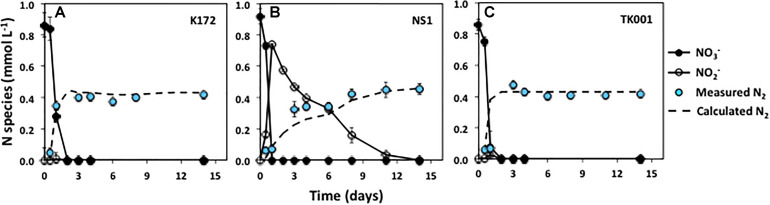
Time series of NO_3_^–^, NO_2_^–^ and measured N_2_ during denitrification with three different denitrifying *Thauera* strains (K172, NS1 and TK001) in batch cultures amended with ∼ 0.9 mmol L^–1^ NO_3_^–^ and acetate. Error bars represent the standard errors for three to four microcosm replicates. The dashed line indicates the N_2_ concentrations calculated assuming decreases in initial NO_3_^–^-N concentrations are completely reduced to N_2_.

Anoxic CSBK medium containing 1 mmol L^–1^ of acetate and NO_3_^–^ (1:1) in sealed serum bottles was amended with different ratios of pre-sterilized solutions of sodium bicarbonate (1 M) and sodium carbonate (1 M). The range of initial pH in these bottles was 7.5 to 10 (set 5; Table 2). The bottles were inoculated with *Thauera* isolates at similar initial cell densities (OD_600_ ≅ 0.005). Headspaces of these bottles were flushed and equilibrated with He to barometric pressure prior to incubation at 30°C. The reduction of NO_3_^–^, production of reduced intermediates (NO_2_^–^, N_2_O) and N_2_ upon denitrification was measured periodically in the aqueous phase and headspace of bottles during 14 days of incubation. The time of incubation was chosen based on the observations from initial denitrification experiments conducted at pH 7.5 (Figure 1). Considering the amount of N_2_ produced in cultures under this pH equivalent to 1, the fold change was calculated using equation 3 and measured concentrations of N_2_ in the bottles. For values lower than 1, the fold decrease was calculated using equation 4.

(3) Foldchange=(1/[N]2)optimum⁢pH*[N]2increased⁢pH

(4) Fold⁢decrease=-1/Fold⁢change

### Quantitative PCR (qPCR) Assay

Culture subsamples (1 mL) from incubations with optimum and higher pH (section “Effect of Initial pH on NO_3_^–^ Reduction to N_2_”) were transferred to 1.5 mL sterilized RNase free microcentrifuge tubes during early and late exponential phases and were centrifuged at 12,000 rpm for 30 min to collect cell pellets. The collected cell pellets were frozen immediately at −80°C. RNA was extracted from these frozen cell pellets within 2 weeks of collection using the RNeasy kit (Qiagen). For extraction, the cell pellets were first re-suspended in 100 μL of TE buffer (10 mM Tris, 1 mM EDTA) containing 15 mg/mL lysozyme (Sigma) and the tubes were incubated at room temperature on a shaker at 300 rpm for 10 min. Buffer RLT (Qiagen) was then added (350 μL) to the tubes followed by vigorous vortexing. The resulting mixture was transferred to a 2 mL tube containing 25–50 mg of acid-washed glass beads (212–300 μm; Sigma) and the cells were disrupted in a Tissuelyser LT (Qiagen) at 50 Hz for 5 min. The lysate obtained was centrifuged at maximum speed for 5–10 s, the supernatant was transferred to a clean tube, and then mixed with 220 μl of 100% ethanol. The extracted RNA was purified from the mixture using RNeasy spin columns according to the manufacturer’s instructions and quantified using a nanophotometer (Implen).

Total RNA (100–500 ng) was reverse transcribed to obtain cDNA using the SuperScript IV VILO Master Mix (Invitrogen) according to the manufacturer’s instructions. The cDNA concentrations of all the samples were normalized to 1 ng/μl with PCR grade water. Real time qPCR assays were performed with primers specific for 16S rRNA, *nirS* (nitrite reductase) and *nosZ* (nitrous oxide reductase) genes using PowerUP SYBR Green Master Mix (Applied Biosystems) on a Quanstudio 3 Real Time PCR System (Thermofisher) running Quantstudio Design and Analysis v1.5.1 Software. The primer sequences used for PCR amplification of these genes are summarized in Table 3. The 16S rRNA gene primers were designed based on the similarity of aligned 1400-1500 bp 16S rRNA sequences of *Thauera* strains used in this study. The PCR cycling conditions were 95°C for 2 min followed by 45 cycles of 95°C for 1 s and 60°C for 30 s. The specificity of the PCR reaction was confirmed using melt curve analysis. Standard curves were performed using control cDNA from representative samples that yielded a dynamic linear range of 0.005 to 50 ng sample per reaction and efficiencies ranging from 96.8 to 102.6% (Bustin et al., 2009). The data was analyzed using the Thermofisher relative quantification application on the Thermofisher cloud that employs the 2^–ΔΔ*CT*^ method for determining relative gene expression (Schmittgen and Livak, 2008).

**TABLE 3:** Sequences of primers used for quantitative PCR (qPCR) and parameters of calibration curves used for qPCR analysis.

Target gene	Amplicon length (bp)	Forward primer	Reverse primer	Amplification efficiencies (%)	*R*^2^	References
16S rRNA	108	GACCTCGCGCGA TTGGAG	CCAGTGTGGCG GATCATCC	102.6	0.999	This study
*nirS*	164	TACCACCCSGARCC GCGCGT	GCCGCCGTCRT GVAGGAA	96.8	0.991	Braker et al., 1998
*nosZ*	259	CGCRACGGCAASAA GGTSMSSGT	CAKRTGCAKSGC RTGGCAGAA	97.6	0.994	Henry et al., 2006

### Gene Sequence Analysis and Nucleotide Accession Numbers

The cDNA amplicons from representative end-point qPCR reactions for the 16S rRNA, *nirS* and *nosZ* genes were purified using PCR spin columns (Sigma). The gene sequences were obtained by Sanger dideoxy sequencing at the Core DNA Services Laboratory of the University of Calgary with both forward and reverse primers (Table 3). The resulting sequences were assembled into contigs using SnapGene software^2^. The gene identity of assembled sequences was confirmed using NCBI Nucleotide BLAST search using 16S rRNA gene sequences as control sequences for the three strains^3^. The identified partial *nirS* and *nosZ* sequences of strains of *Thauera* obtained were submitted to NCBI GenBank database (accession numbers MT186692 to MT186694 and MT186689 to MT186691, respectively).

## Results

### Denitrification Kinetics of *Thauera* Strains

All of the tested *Thauera* strains rapidly reduced NO_3_^–^ to N_2_ using acetate as an electron donor upon incubation under identical initial conditions (∼ 0.9 mmol L^–1^ NO_3_^–^, pH 7.5; Figure 1). Incubations were performed in four replicates via two separate experiments (both included in Set 1; Table 2) conducted with two replicates in each. The rates of denitrification, and end products were similar within the replicates and the experiments for each strain. Nitrate concentrations were reduced to below detection levels (< 0.001 mmol L^–1^) in all the cultures within two days.

The denitrification kinetics in cultures with K172 and TK001 were similar, with complete reduction of NO_3_^–^ to N_2_ occurring within three days of incubation for both cultures (with denitrification rates of 0.431 and 0.429 mmol L^–1^ d^–1^, respectively; Table 4). On the other hand, NS1 took 14 days for complete conversion of NO_3_^–^ to N_2_, with transient accumulation of NO_2_^–^ concentrations that peaked within two days at 0.74 mmol L^–1^ and declined over the course of incubation. The denitrification rate (0.071 mmol L^–1^ d^–1^) of NS1 was also lower as compared to the other two strains. No significant accumulation of N_2_O was detected in any of these cultures, and the final concentrations of 0.42 to 0.45 mmol L^–1^ of N_2_ indicated that 97.3 to 98.9 % of the initial NO_3_^–^-N was reduced to N_2_ (Figure 1 and Table 4).

**TABLE 4:** Microcosm parameters, microbial growth characteristics (first order growth rate), denitrification rate and N_2_ production by denitrifying *Thauera* strains amended with NO_3_^–^ and acetate in CSBK medium in 14-day microcosm incubations at 30°C (Figure 1).

Strain	Initial NO_3_^–^ concentrations^a^ (mmol L^–1^)	Final concentrations			Denitrification Rate (mmol L^–1^ d^–1^)	Cell growth	
		N_2_	% recovered	Yield coefficient		Maximum	Exponential growth rate
		(mmol L^–1^)^a^	as N_2_^b^	(Y_*P/S*_)^c^		OD_600_^a^	(d^–1^)^a^
K172	0.862 (0.077)	0.420 (0.008)	97.45	0.431	0.431	0.119 (0.002)	0.697 (0.078)
NS1	0.918 (0.050)	0.454 (0.034)	98.91	0.427	0.071	0.095 (0.005)	0.592 (0.011)
TK001	0.859 (0.035)	0.418 (0.004)	97.32	0.418	0.429	0.042 (0.002)	0.321 (0.040)

^a^ Numbers in parentheses are standard errors for three to four replicates. ^b^ % N_2_ recovered = (Total N_2_ produced in culture bottles/(0.5*Initial nitrate))*100. ^c^Y_*P/S*_ = (P_*t*_−P_0_)/(S_0_−S_*t*_) where P_0_ and P_*t*_ are product concentrations at time 0 and final, respectively; S_0_ and S_*t*_ are substrate concentrations at time 0 and final substrate concentrations, respectively.

The microcosms that were prepared using denitrification intermediates as electron acceptors (NO_2_^–^ and N_2_O) instead of NO_3_^–^ also gave similar rates of N recovery as N_2_, with no N_2_O detected as an intermediate in NO_2_^–^ amended cultures (Supplementary Figure 2). However, the rates of reduction of these intermediates were lower than those with NO_3_^–^. There was a lag period of 0.5 to 3 days before the *Thauera* strains initiated reduction of NO_2_^–^ and N_2_O. On average, 0.42 ± 0.01 mmol L^–1^ of N_2_ was produced in these cultures by the end of the incubation period (Supplementary Figure 2).

The bacterial cell growth characteristics and product yield coefficients for the three *Thauera* strains are summarized in Table 4. The strain K172 cultures consumed 0.86 mmol L^–1^ NO_3_^–^ and grew to a highest maximum OD_600_ of 0.119. In comparison, NS1 and TK001 grew to a lower maximum OD_600_ of 0.095 and 0.042, respectively after similar amounts of NO_3_^–^ consumption (i.e., concentrations of 0.85 and 0.91 mmol L^–1^, respectively; Table 4). The rates of denitrification observed in the microcosms were inversely correlated with the exponential growth rates of these strains. The rate of NO_3_^–^ reduction was highest in TK001 cultures with lowest exponential growth rate of 0.321 d^–1^. Strain NS1 showed lowest rate of NO_3_^–^ reduction but had higher exponential growth rate of 0.592 d^–1^. Although the K172 cultures had a rates of NO_3_^–^ reduction similar to that of TK001, their exponential growth rates were highest among the three strains (0.697 d^–1^; Table 4 and Supplementary Figure 3). The product yield coefficients from the cultures were 0.42 to 0.43 (Table 4).

### Effect of Initial NO_3_^–^ Concentrations on Denitrification Products

**FIGURE 2:**
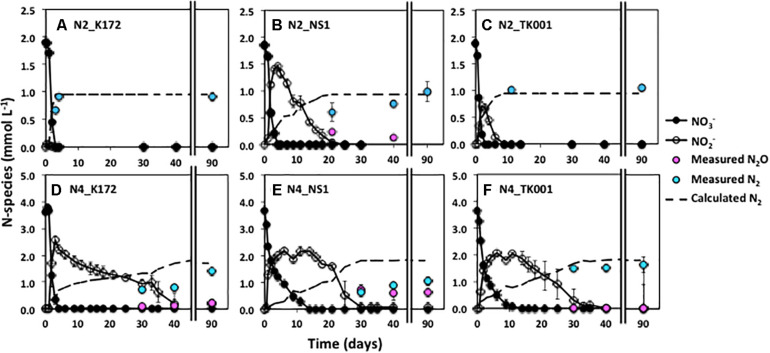
Time series of NO_3_^–^, NO_2_^–^, and measured N_2_ during denitrification with three different denitrifying *Thauera* strains (K172, NS1 and TK001) in batch cultures amended with two different initial NO_3_^–^ concentrations of ∼ 2 mmol L^–1^ (N2; upper graphs) or ∼ 5 mmol L^–1^ (N4; lower graphs). Error bars represent the standard errors for three to four replicates. The dashed line indicates the N_2_ concentrations calculated assuming decreases in initial NO_3_-N concentrations are completely reduced to N_2_.

All three bacterial strains were further cultivated on two to five-fold higher NO_3_^–^ concentrations (1.90 to 4.60 mmol L^–1^; set 2; Tables 2, 5) with equimolar initial acetate concentrations. Varying the initial inputs of NO_3_^–^ in culture bottles affected the denitrification rate and abundance of intermediate products. Complete reduction of NO_3_^–^ to N_2_ was observed in all three cultures amended with 2.0 mmol L^–1^ initial NO_3_^–^. The percent N_2_ recovered from these bottles ranged from 96.6 to 110.9 % (Table 5). Denitrification was complete after 5 (K172), 60 (NS1) and 11 (TK001) days of incubation. Nitrite accumulated at higher concentrations (up to 1.46 mmol L^–1^) and persisted longer in the NS1 cultures as compared to the other two strains. The formation of N_2_O upon reduction of NO_2_^–^ was not detected in cultures with K172 and TK001 while up to 0.23 mmol L^–1^ N_2_O formed upon reduction of NO_2_^–^ in the NS1 cultures (Figure 2).

**TABLE 5:** Production and distribution of reduced products of denitrification coupled to acetate oxidation in batch cultures of *Thauera* strains amended with different NO_3_^–^ concentrations.

Strain	Initial	Incubation	Residual	Maximum	Maximum	Final reduced products^a^			% recovered as
	NO_3_^–a^	time	NO_3_^–^	NO_2_^–^ produced	N_2_O produced	(mmol L^–1^)			N_2_^b^
	(mmol L^–1^)	(days)	(mmol L^–1^)	^a^(mmol L^–1^)	^a^(mmol L^–1^)						
						NO_2_^–^	N_2_O	N_2_	
K172	0.86 (0.08)	14	ND	0.01 (0.04)	ND	ND	ND	0.42 (0.01)	97.45
	1.90 (0.10)	90	ND	0.05 (0.05)	ND	ND	ND	0.92 (0.05)	96.64
	2.87 (0.08)	90	ND	1.20 (0.07)	ND	ND	ND	1.41 (0.01)	98.40
	3.62 (0.07)	90	ND	2.56 (0.11)	0.23 (0.00)	0.20 (0.04)	0.23 (0.03)	1.41 (0.09)	82.46
	4.60 (0.18)	90	ND	3.19 (0.12)	0.20 (0.05)	0.34 (0.07)	0.20 (0.04)	1.66 (0.25)	77.93
NS1	0.92 (0.05)	14	ND	0.74 (0.01)	ND	ND	ND	0.45 (0.03)	98.91
	1.85 (0.06)	90	ND	1.46 (0.02)	0.23 (0.00)	ND	ND	0.98 (0.18)	105.95
	2.78 (0.01)	90	ND	2.05 (0.06)	0.50 (0.00)	ND	0.44 (0.00)	0.80 (0.18)	57.84
	3.67 (0.09)	90	ND	2.18 (0.08)	0.72 (0.20)	0.06 (0.08)	0.63 (0.09)	1.05 (0.14)	58.17
	4.57 (0.01)	90	ND	2.64 (0.12)	0.80 (0.11)	0.43 (0.12)	0.80 (0.12)	0.85 (0.13)	41.06
TK001	0.86 (0.03)	14	ND	0.02 (0.01)	ND	ND	ND	0.42 (0.00)	97.37
	1.88 (0.07)	90	ND	0.70 (0.02)	ND	ND	ND	1.04 (0.00)	110.90
	2.77 (0.07)	90	ND	1.55 (0.02)	ND	ND	ND	1.49 (0.10)	107.20
	3.64 (0.05)	90	ND	2.06 (0.12)	0.01 (0.00)	ND	0.01 (0.00)	1.52 (0.13)	83.60
	4.49 (0.20)	90	ND	2.62 (0.27)	0.05 (0.00)	ND	0.05 (0.00)	1.75 (0.01)	78.06

^a^ Numbers in parentheses are standard errors for three to four replicates. ^b^ % N_2_ recovered = (Total N_2_ produced in culture bottles/(0.5*Initial nitrate))*100. ND, not detected; detection limit for NO_3_^–^, NO_2_^–^ and N_2_O is 0.001 mmol/L.

On average, 0.98 ± 0.06 mmol L^–1^ N_2_ was produced upon reduction of 1.88 ± 0.03 mmol L^–1^ NO_3_^–^ in all cultures (Table 5). The complete conversion of NO_3_^–^ to N_2_ was further observed up to 2.82 ± 0.07 mmol L^–1^ NO_3_^–^ in cultures with strains K172 and TK001 but not in cultures with NS1. There was transient accumulation of 1.20 to 1.55 mmol L^–1^ NO_2_^–^ only as an intermediate in the K172 and TK001 cultures. In contrast, NO_2_^–^ and N_2_O both accumulated in NS1 incubations. At the end of a 90-day incubation period, NO_2_^–^ was completely reduced and 0.44 ± 0.00 mmol L^–1^ N_2_O remained with the formation of 0.80 ± 0.18 mmol L^–1^ N_2_ in all cultures (Table 5).

The percentage of NO_3_^–^-N recovered as N_2_ at the end of a 90-day incubation decreased as initial NO_3_^–^ concentrations increased. The decrease was similar in cultures with K172 and TK001. A four to five-fold higher input of NO_3_^–^ (e.g., than 1 mmol L^–1^) in these cultures gave 14.38 to 19.42 % lower N_2_ (Table 5 and Figures 1, 2). A larger decrease in N_2_ production occurred with strain NS1, ranging from 40.74 to 57.85 %, and the decrease was observed at comparatively lower fold increase in initial NO_3_^–^ inputs. Average initial NO_3_^–^ concentrations of 3.65 and 4.59 mmol L^–1^ NO_3_^–^, produced NO_2_^–^, N_2_O and N_2_ at the end of 90-day incubation in cultures with K172 and NS1. In the TK001 cultures amended with similar NO_3_^–^ concentrations, only N_2_O and N_2_ were formed (Table 5 and Figures 2C,F). Overall, the reduction of NO_3_^–^ to N_2_ was less complete when higher NO_3_^–^ concentrations were added in the tested *Thauera* cultures.

### Nitrite Inhibition of *Thauera* Strains as a Function of NO_2_^–^ Concentrations

High concentrations of NO_2_^–^ can be toxic to bacteria, thus inhibiting their metabolic activities. In order to investigate the effects of NO_2_^–^ concentrations on the denitrification potential of the *Thauera* strains, two sets of kinetic experiments (both included in Set 3; Table 2) were conducted using NO_2_^–^as the sole electron acceptor when added at different concentrations (1 to 5 mmol L^–1^) and with equimolar initial concentrations of acetate as an electron donor (i.e., in 1:1 ratio). The patterns of NO_2_^–^ reduction observed were similar in both the experiments. However, the rates of NO_2_^–^ reduction were distinct among the three *Thauera* strains and different starting NO_2_^–^ concentrations in batch cultures. With ∼1 mmol L^–1^ of initial NO_2_^–^, the reduction started after 2 (K172), 1 (NS1) and 0.5 (TK001) days of incubation of cultures (Supplementary Figure 2). The lag period before the initiation of NO_2_^–^ reduction in these cultures further increased with the increase in NO_2_^–^ concentration. For instance, it took 5 (K172), 3 (NS1) and 0.7 (TK001) days for *Thauera* strains to start reducing NO_2_^–^ in cultures with five-fold higher NO_2_^–^ (∼5 mmol L^–1^; Supplementary Figure 4). The NO_2_^–^ concentrations had minimal effects on the NO_2_^–^ reduction potential of strain TK001 in the beginning of incubation as compared to the other two *Thauera* strains. However, after 15 days of incubation there were no significant changes in the amounts of NO_2_^–^ reduced in these cultures (Supplementary Figure 4C). Similarly, there was not much NO_2_^–^ reduction in cultures with strain K172 after a similar incubation period (18 days; Supplementary Figure 4A). In comparison, levels of NO_2_^–^ became more constant in cultures with strain NS1 in a shorter time of incubation (after 10 days; Supplementary Figure 4B). Continued incubation of all the *Thauera* cultures for 30 days did not show any additional substantive changes in NO_2_^–^ reduction after these time periods.

**FIGURE 3:**
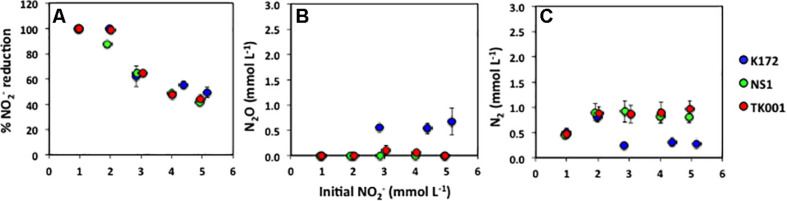
**(A)** Percent NO_2_^–^ reduction (Equation 2), and final concentrations of **(B)** N_2_O and **(C)** N_2_ produced after 30 days of incubation in batch cultures of *Thauera* strains, shown as a function of varying initial NO_2_^–^ concentrations (∼ 1 to 5 mmol L^–1^; *x*-axis). Error bars represent standard errors for three to four replicates. The time series for these batches are shown in Supplementary Figure 4.

Nitrite was completely reduced to N_2_ in all the cultures with initial NO_2_^–^ concentrations of ∼1 mmol L^–1^ (Supplementary Figure 2). However, in cultures with higher initial NO_2_^–^ concentrations complete reduction of NO_2_^–^ to N_2_ was observed only in cultures with strain K172 and 2 mmol L^–1^ NO_2_^–^ (Supplementary Figure 4A). The percent reduction of NO_2_^–^ decreased with an increase in the initial NO_2_^–^ concentrations with strain NS1 being most affected (Figure 3A). With a 5-fold increase in NO_2_^–^ concentrations, the percent NO_2_^–^ reduction in cultures decreased to 49.21% (K172), 41.39% (NS1) and 43.93% (TK001). The reduction of NO_2_^–^ produced only N_2_ in the cultures with NS1 (Figures 3B,C). Nitrous oxide was not detected at any time point during incubation of these cultures. However, both N_2_O and N_2_ were produced as reduced end products of NO_2_^–^ reduction in cultures with K172 and TK001. More N_2_O (0.56 to 0.67 mmol L^–1^) was produced in K172 cultures than in TK001 cultures (0.06 to 0.15 mmol L^–1^ of N_2_O; Figure 3B). Similar amounts of N_2_ were produced in cultures with NS1 (0.46 to 0.92 mmol L^–1^) and TK001 (0.47 to 0.96 mmol L^–1^). The N_2_ produced in K172 cultures were much lower in comparison (0.24 to 0.80 mmol L^–1^) especially at high initial NO_2_^–^ concentrations (> 2 mmol L^–1^; Figure 3C). Overall, an average of 2.18 ± 0.36 (K172), 1.88 ± 0.16 (NS1) and 2.02 ± 0.11 (TK001) mmol L^–1^ of NO_2_^–^ was reduced to N_2_O and N_2_ in cultures. The average increase in alkalinity of these cultures was 1.58 ± 0.10 meq/L caused by production of dissolved CO_2_ upon oxidation of acetate coupled to NO_2_^–^ reduction. The culture pH increased to 8.14 ± 0.11 from 7.50.

### Effect of High pH on Completeness of Denitrification

pH is a measure of the hydrogen ion concentration in the aqueous phase and alkalinity is a measure of the capacity of aqueous phase in a contained system to neutralize acids. In anoxic closed carbonate system such as the culture bottles in our experiments, changes in dissolved inorganic species such as bicarbonates (HCO_3_^–^) and carbonates (CO_3_^2–^) upon acetate oxidation to CO_2__(__*g*__)_ cause changes in alkalinity and pH unless there are mineral buffers. At the end of batch experiments that were initially amended with ∼1 mmol L^–1^ acetate and NO_3_^–^, a decrease of 0.72 ± 0.03 mmol L^–1^ in acetate concentration was observed. Based on the redox stoichiometry (equation 5), 0.55 ± 0.02 mmol L^–1^ (76.4 ± 4.7%) of acetate was oxidized for complete reduction of NO_3_^–^ to N_2_ and the remaining 0.18 ± 0.06 mmol L^–1^ (25.0 ± 7.0 %) was presumably incorporated into bacterial biomass and other by-products (Figure 1 and Supplementary Figure 5; Chen et al., 2017).

(5) 0.625CHCOO3+-NO+3-0.375H→+0.5N+21.25CO2+1.75⁢H⁢O2

**FIGURE 4:**
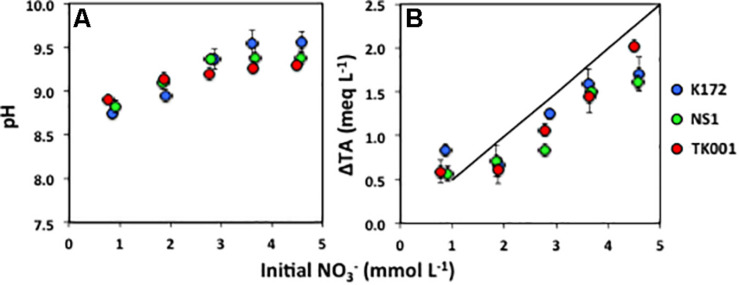
**(A)** Final pH and **(B)** change in total alkalinity (ΔTA; meq L^–1^) expressed as a function of initial NO_3_^–^ concentrations (mmol L^–1^) (Table 4) produced through acetate oxidation coupled to denitrification by *Thauera* strains in batch cultures. Error bars represent standard errors for three to four replicates.

Although carbon dioxide (CO_2__(g)_) was not detected or detected at negligible concentrations by GC analysis, the CO_2_ loss to the atmosphere is negligible given the sealed culture bottles used. This indicates that all CO_2_ produced remained in the aqueous phase, where it would partition into the dissolved inorganic species. Accordingly, the pH of these cultures increased from 7.50 to 8.82 ± 0.08 (Figure 4). Using the measured pH values and dissociation constants, the total alkalinity was calculated to increase from 2.51 to 3.00 meq/L (Supplementary Material section 3). The measured alkalinity values (3.26 ± 0.16 meq/L) in these cultures upon completion of denitrification by *Thauera* strains were very similar to the calculated values and the reaction is well constrained for the lower initial NO_3_^–^ concentrations (∼1 mmol L^–1^).

The increase in pH and alkalinity of cultures with increasing initial NO_3_^–^ concentrations is shown in Figure 4. The cultures amended with higher initial NO_3_^–^ concentrations tended to not produce as high alkalinity as equation 5 would predict (Figure 4). The difference was highest at ∼ 5 mmol L^–1^ initial NO_3_^–^ concentrations especially in the cultures with strains K172 and NS1. In comparison, at the similar initial NO_3_^–^ concentrations, the difference was lower in the cultures with TK001 that showed comparatively more complete denitrification (Table 4). This could be due to incomplete denitrification caused by high pH (up to 9.56), and less production of CO_2_ in NS1 and K172 cultures.

### N_2_O Reduction Kinetics as a Function of Increasing pH

**FIGURE 5:**
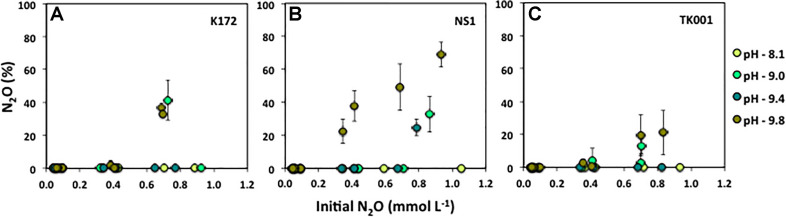
Percent N_2_O_*REMAINING*_ calculated as ([N_2_O]/[N_2_O + N_2_])*100 after 15 days incubation of batch cultures of three different *Thauera* strains (K172, NS1 and TK001) as a function of varying initial N_2_O concentrations (0.05 to ∼ 1.0 mmol L^–1^), and varying initial pH values (8.10 to 9.80). Error bars represent standard errors for two replicates.

The direct effect of initial pH on growth and reduction of N_2_O by *Thauera* strains was tested in batch cultures with 0.04 to 1 mmol L^–1^ N_2_O as the sole electron acceptor and 0.5 mmol L^–1^ acetate as the electron donor (set 4; Table 2). The average amounts of remaining N_2_O in the cultures after 15 days of incubation are summarized in Figure 5. The percent N_2_O is plotted against the initial N_2_O concentrations added to the cultures. The reduction of N_2_O to N_2_ was not much affected at pH values of ∼8.1 where N_2_O was completely reduced to N_2_ at all concentrations in cultures with *Thauera* strains except in cultures with 1.05 mmol L^–1^ of initial N_2_O and inoculated with strain NS1. Minimal amounts of N_2_O (0.02 ± 0.04 %) remained in these cultures (Figure 5B). With the increase in initial pH, the remaining N_2_O that was not reduced to N_2_ increased in the cultures when 0.79 to 1 mmol L^–1^ initial N_2_O was provided. The percent N_2_O remaining was 32.73 ± 10.81, 24.72 ± 5.14 and 68.97 ± 7.63 % in NS1 cultures at pH 9.00, 9.40, and 9.80, respectively. Nitrous oxide added at lower concentrations (0.04 to 0.10 mmol L^–1^) to these cultures was not detected at any of the pH conditions tested and was completely reduced to N_2_. At the highest tested pH 9.8, the percent N_2_O not utilized increased from 22.49 ± 7.45 to 68.97 ± 7.63 % with the increase in initial added N_2_O from 0.35 to 0.93 mmol L^–1^ (Figure 5B). Similarly, at pH 9.8, the percent N_2_O not utilized increased with the increase in initial N_2_O concentrations in cultures with strains K172 and TK001, however, the amounts of increases were lower as compared to those in cultures with strain NS1. The increase in remaining %N_2_O cultures was from 0.29 to 36.97 % (K172) and 0.58 to 21.18 % (TK001) at initial N_2_O concentrations ranging from 0.35 to 0.83 mmol L^–1^ (Figures 5A,C). No significant amounts of remaining N_2_O were detected in cultures with K172 and TK001 at pH 9.4 at any of the added N_2_O levels. However, up to 41.29 ± 12.15 % (K172) and 12.80 ± 4.63 % (TK001) N_2_O remained in cultures at pH 9.0 and initial N_2_O concentrations of more than 0.40 mmol L^–1^. Overall, at higher pH, the percent reduction of high concentrations of N_2_O to N_2_ was lower than at pH 7.5 and 8.1 (Figure 5).

### Abundance of Denitrification Genes and N_2_ Production Under High pH Conditions

The expression of denitrification genes (*nirS* and *nosZ*; equation 6) was tested using reverse transcription qPCR (RT-qPCR) analysis to determine the role of these genes in prolonged transient accumulation of denitrification intermediates (NO_2_^–^ and N_2_O) under high pH conditions.


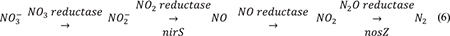


**FIGURE 6:**
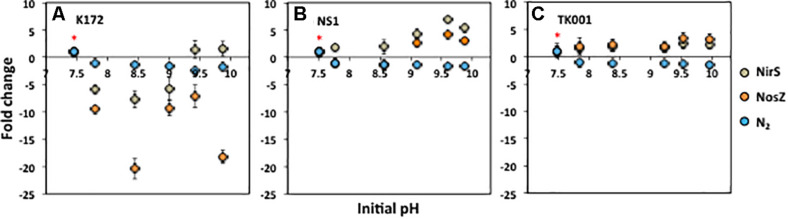
Changes in production of N_2_ and expression of denitrification genes (NirS and NosZ) as a function of increasing pH in batch cultures of denitrifying *Thauera* strains (K172, NS1 and TK001) amended with 1 mmol L^–1^ of NO_3_^–^ and acetate. The red star (*) indicates optimum pH-7.5 taken as equivalent to 1 for comparison with high pH conditions. Error bars represent standard errors for three replicates.

The reduction of NO_3_^–^, production of N_2_ and expression of these genes was quantified in a separate experiment conducted at pH values identical to those measured in the cultures of denitrifying *Thauera* strains (Figures 4, 6). After 15 days of incubation, NO_3_^–^ was completely reduced to NO_2_^–^ in all the cultures except K172 cultures with pH of 9.42 and 9.87 (Supplementary Figure 6). The NO_2_^–^ formed was further reduced completely in cultures with TK001 but not in cultures with K172 and NS1. The remaining NO_2_^–^ concentrations were 5 to 6 fold lower (0.02 to 0.05 mmol L^–1^) in cultures inoculated with K172 than those with NS1 (0.13 to 0.26 mmol L^–1^). Nitrous oxide was not detected in any of the TK001 cultures throughout the incubation period, while minimal amounts of N_2_O (< 0.001 mmol L^–1^) were produced in cultures with NS1 and pH 9.88. Comparatively higher amounts of N_2_O (0.02 to 0.24 mmol L^–1^) accumulated in cultures with K172. The concentrations of NO_2_^–^ and N_2_O in cultures of both these strains (K172 and NS1) correlated with their pH values. Increased pH values were associated with increased concentrations of these intermediates detected in these cultures.

For quantification of *nirS* and *nosZ* genes, the 16S rRNA gene was used as a control and their relative abundances at higher pH values were compared to those at optimum pH conditions using the 2^–ΔΔ*CT*^ method (Schmittgen and Livak, 2008). The standard curves for qPCR calibration were linear (*R*^2^ = 0.99) for all the three genes with amplification efficiencies between 96.8 to 102.6 % (Table 3). The amounts of N_2_ produced decreased with an increase in pH of cultures with the three *Thauera* strains; up to a 2.40, 1.66 and 1.48-fold decrease was observed for cultures inoculated with K172, NS1 and TK001, respectively as compared to the cultures with optimum pH (Figure 6). The decrease was most evident in the cultures exhibiting pH values between 9.11 and 9.90. The RT-qPCR analysis of *nirS* and *nosZ* transcripts supported this decrease of N_2_ production in batch cultures with K172 (Figure 6A). The *nirS* transcription levels in these cultures were 5.79 to 7.72-fold lower than the control cultures except for the cultures having pH values of 9.42 and 9.87. The abundance of *nirS* in these cultures increased by 1.40 to 1.59-fold as compared to its abundance at optimum pH conditions. The higher pH levels more greatly impacted the transcription levels of *nosZ* as its abundance decreased by 7.13 to 20.38-fold in these cultures (Figure 6A). Note that the samples for qPCR from K172 cultures were taken during the exponential growth phases; *t* = 2 and *t* = 5 to 15 days for optimum pH and higher pH conditions, respectively.

The bacterial cells for RT-qPCR were collected at the same time, *t* = 5 and *t* = 15 days, for cultures inoculated with strains TK001 and NS1, respectively. Both *nirS* and *nosZ* were highly expressed in the cultures with NS1 and TK001 at all high pH conditions despite less N_2_ production (Figures 6B,C). For culture NS1, the *nirS* transcription levels were 1.77 to 6.86-fold higher in the cultures containing increased pH compared to the optimum pH control. The *nosZ* transcription levels also followed the similar pattern except in cultures having pH values of 7.77 and 8.56. In fact, the abundance of *nosZ* decreased by 1.10 to 1.39-fold in these cultures (Figure 6B). In all TK001 cultures incubated under all conditions, *nosZ* was more abundantly transcribed than *nirS*. However, the abundance of both these genes was higher under higher pH conditions than at the control pH. The transcription levels of *nirS* and *nosZ* were 1.61 to 2.44 and 1.83 to 3.30-fold higher, respectively, in these cultures (Figure 6C). The observed difference in the expression patterns and transcription levels of *nirS* and *nosZ* in cultures with three *Thauera* strains may be attributed to differences in their denitrification kinetics, sensitivity to pH stress conditions, and sampling times for analysis.

## Discussion

### Type and Concentration of N-Sources Affect Denitrification Regulatory Phenotypes

This study focused on the effects of physico-chemical parameters of the microbial growth environment on the metabolic activities of denitrifying bacterial isolates affiliated to the genus *Thauera*, namely *T. aromatica* K172, *T. aromatica* NS1 and *T. aminoaromatica* TK001. The biokinetic experiments conducted at 30°C and under anoxic conditions showed denitrification differences between these strains. Strains of *Thauera* previously studied were grouped into two categories based on the observed differences in their denitrification regulatory phenotypes (DRPs; Liu et al., 2013). The strains were characterized either by rapid, complete onset (RCO) or by progressive onset (PO) of denitrification genes linked to NO_3_^–^ reduction and accumulation of intermediates like NO_2_^–^ (Liu et al., 2013). At lower NO_3_^–^ concentrations (∼1 mmol L^–1^), strains K172 and TK001 displayed an RCO type of DRP with non-detectable NO_2_^–^ and N_2_O accumulation during denitrification coupled with acetate oxidation (Figures 1A,C). However, strain S2 studied by Liu et al. (2013), and similar to strain TK001, was reported to have a PO type of DRP upon reduction of NO_3_^–^ (2 mM per 50 mL) coupled to acetate oxidation. The observed differences between the two *T. aminoaromatica* strains (TK001 and S2) in these two independent studies seem to be less related to similar culture conditions used than their reported metabolic activities. Even though there are high 16S rRNA gene sequence similarities (99% sequence identity), considerable phenotypic differences are known to exist between these two strains (Mechichi et al., 2002; Fida et al., 2016). The type strain K172 that showed similar denitrification phenotypic traits as TK001 in this study was also revealed to be genotypically different in its protein profile compared to strain S2 (Mechichi et al., 2002). The variation in observed DRPs of these strains could possibly be related to the expression patterns of proteins and abundance of denitrification genes (Mechichi et al., 2002; Liu et al., 2013). In contrast, strain NS1 exhibited similarities to a PO type of DRP and transient accumulation of NO_2_^–^ resulting from reduction of NO_3_^–^ (Figure 1B). This strain is phylogenetically more related to strains K172 and S100 (> 90% 16S rRNA gene similarity; Suri et al., 2019) but unexpectedly showed lower denitrification rates as compared to the type strain K172 under identical denitrifying culture conditions.

At higher NO_3_^–^ concentrations (> 2 mmol L^–1^), the denitrification kinetics of the three *Thauera* strains became more similar showing a PO type of DRP and prolonged accumulation of NO_2_^–^ and N_2_O (Figure 2 and Table 4), even at an ideal temperature (30°C). Suri et al. (2017) reported incomplete NO_3_^–^ reduction and persistence of relatively high concentrations of NO_2_^–^ formed through reduction of similarly high NO_3_^–^ concentrations under these conditions. The toxicity posed by produced NO_2_^–^ to dominant *Thauera* and *Pseudomonas* species was thought to be the reason but was not evaluated. In the present study, the NO_2_^–^ accumulated to maximum concentration of 3.2 mmol L^–1^ in microcosms (Table 4). The exposure of *Thauera* strains to NO_2_^–^ concentrations (0.9 to 5.2 mmol L^–1^), even higher than the accumulated concentrations (0.01 to 3.19 mmol L^–1^), did not appear to be inhibitory (Supplementary Figure 4). Instead, the denitrification rates were slower and ceased after reduction of 1.9 mmol L^–1^ NO_2_^–^. The use of NO_2_^–^ as compared to NO_3_^–^ as a sole initial electron acceptor had a variable impact on the DRPs. Strains TK001 and NS1 behaved similarly showing a RCO type of DRP but K172 showed a PO type of DRP. Strain K172 only partially reduced NO_2_^–^ to N_2_ producing N_2_O as a product in addition to N_2_ (Supplementary Figures 2, 4 and Figure 3). In contrast, Liu et al. (2013) showed N_2_O produced as the only end product by *T. phenylacetica* characterized to have PO type of DRP. Considerable genotypic differences exist between these tested species, namely *T. aromatica* and *T. phenylacetica*, that appeared to be a reason for observed differences in their DRPs (Suri et al., 2019).

### Increased pH Affects Denitrification End Products

The impaired reduction of NO_3_^–^ and the produced intermediate NO_2_^–^ by *Thauera* strains under the influence of increasing temperature and decreasing electron donor concentrations has previously been shown in the laboratory (Fida et al., 2016; Suri et al., 2017). At temperatures above 45°C and at NO_3_^–^ concentrations equivalent to the 1 mmol L^–1^ concentration used in this study, denitrification halts at NO_2_^–^ (Reinsel et al., 1996; Greene et al., 2003; Fida et al., 2016; Okpala et al., 2017). However, subsequent complete reduction of NO_2_^–^ to N_2_ occurs at temperatures below 45°C provided sufficient amounts of preferable electrons donors are available (Voordouw et al., 2009; Fida et al., 2016; Okpala et al., 2017). Similar patterns of NO_2_^–^ persistence during NO_3_^–^ reduction can also occur with an increase in salinity from 0.5 to 2.5 M (An et al., 2017). Here, we additionally demonstrate that pH changes, particularly alkalinity, can cause substantive impact on denitrification. The assays performed demonstrate that the denitrification product ratios (NO_2_^–^: N_2_O: N_2_) can vary with the variation in pH (Figure 4 and Table 5).

Higher pH conditions (pH ≥ 9) had a greater impact on the production and reduction of NO_2_^–^ and N_2_O compared to the use of high NO_3_^–^ concentrations (> 2 mmol L^–1^) by *Thauera* species. Such a pH effect has also been reported in other studies. At a pH of 8 and higher, the growth of *Dechloromonas aromatica* strain RCB was inhibited and there was accumulation of substantial amounts of N_2_O during NO_3_^–^ reduction (Han et al., 2019). In contrast, transient to permanent accumulation of NO_2_^–^ and N_2_O have been observed at neutral to acidic pH conditions. For instance, rates of denitrification in *Thauera*, *Pseudomonas* and *Paracoccus* cultures were lower, with increasing accumulated concentrations of NO_2_^–^ and N_2_O at pH values of 6.0 to 7.5 (Bergaust et al., 2010; Suri et al., 2017; Kim et al., 2017). In this present study, the accumulation of NO_2_^–^ at pH 7.5 was observed only for strain NS1 but was further reduced to N_2_ (Figure 1).

Microbial metabolisms vary with the type and concentration of electron donor, which can substantially affect pH and alkalinity (Gallagher et al., 2012). Acetate is a highly degradable electron donor readily available in many natural environments and its use in this study contributed to pH increase (Figure 4). Denitrifiers like *Thauera* and *Pseudomonas* also possess abilities to use other organic electron donors for driving their metabolism and energy processes (Agrawal et al., 2012; Suri et al., 2017; An et al., 2017). The effect of pH increase on NO_3_^–^ reduction could be mitigated by using electron donors that do not cause pH shifts upon oxidation. Nonetheless, the use of acetate in the current study helped to gain initial knowledge on the overall variable effect of pH on the outcomes of denitrification.

### Increased pH Affects Denitrification Enzyme Activity

The key enzymes that catalyze the reduction of NO_2_^–^ to N_2_O and of N_2_O to N_2_ are nitrite reductase and nitrous oxide reductase, respectively. These enzymes are encoded by the *nirS* (nitrite reductase) and *nosZ* (nitrous oxide reductase) genes in many *Thauera* species and are intracellular but can undergo considerable changes in response to fluctuations in pH and other environmental conditions (Zumft, 1997; Wilks and Slonczewski, 2007; Fida et al., 2016). The mechanisms can include altered enzyme synthesis, inhibition of enzyme function and/or reduced or delayed transcription of denitrification genes (Bergaust et al., 2010; Liu et al., 2010; Fida et al., 2016). Our results indicated a correlation of high pH with reduced transcription of the *nirS* (at pH 7.8-9.0) and *nosZ* (at pH 7.8-9.9) genes resulting in less complete denitrification and lower N_2_ production in cultures of strain K172. Unexpectedly, even though the transcriptional levels of *nirS* were higher at pH ≥ 9, the amount of N_2_O produced from reduction of NO_2_^–^ was much lower than that produced at pH ≤ 9 (Figure 6A, and Supplementary Figure 6A). Nitrate was not completely reduced in these cultures. Since NO_3_^–^ is a preferred electron acceptor compared to NO_2_^–^ and N_2_O, one possible explanation for our observation is that nitrate reductase (encoded by the *nar* gene and not quantified in this study) was still active and can compete more efficiently for electrons than *nirS*, thus causing partial denitrification (Almeida et al., 1995; Liu et al., 2013). The protons from the inside of cytoplasmic membrane are used for NO_3_^–^ reduction while those from the periplasmic side of cytoplasmic membrane are used for NO_2_^–^ reduction. At pH ≥ 9, protons may be comparatively scarce in the periplasm than at pH ≤ 9, thus resulting in inhibition of enzyme activity of nitrite reductase even though *nirS* transcriptional levels were high (Meijer et al., 1979; Glass and Silverstein, 1998). In contrast, the transcriptional levels of *nirS* increased only after depletion of NO_3_^–^ in cultures of *Thauera terpenica* (Liu et al., 2013).

The transcriptional levels of *nirS* and *nosZ* were similar in cultures of strain TK001 at a tested pH, which is characterized to have the RCO type of DRP (Figures 6C, 1). The levels and expression patterns of the *nirS* and *nosZ* genes in cultures of *Thauera* sp. 63 with an RCO type of DRP were also shown to be similar during denitrification at pH 7.5 (Liu et al., 2013). The transcriptional levels of both these genes increased similarly at the beginning of denitrification, reached similar maximum levels and then decreased at the end of denitrification, however, the abundances of *nosZ* transcripts were sustained at higher levels than *nirS* transcripts, similar to our experiments (Liu et al., 2013; Figure 6C). Both *nirS* and *nosZ* were expressed simultaneously at each pH; however, the timing of maximum transcription was delayed with the increase in pH of cultures. This observation suggested that a decrease in the rates of denitrification at pH > 7.5 were caused by slower enzyme activity in comparison to that observed at pH 7.5, as was evident from lower N_2_ production at the incubation time assayed (*t* = 5 days; Figure 6C). Similar patterns of increased transcriptional levels of *nirS* and *nosZ* with the increase in pH were observed in NS1 cultures. Nitrite accumulated in these cultures, an expected effect of the PO type of DRP of this strain (Supplementary Figures 1, 6B and Figure 6). The temporary shut down of electron flow to nitrite reductase, deficiency of co-factors such as Cu required for its activity, inhibition of translation of its mRNA and/or repression of its activity at high pH and high NO_2_^–^ concentrations can also cause NO_2_^–^ to accumulate (Stouthamer, 1991; Baumann et al., 1997; Granger and Ward, 2003). Since there was sufficient electron donor (acetate) present, the observed inhibited NO_2_^–^ reduction can possibly be explained by inhibition of nitrite reductase activity and DRP of the strain. The minimal N_2_O accumulation observed can be explained by the DRP of this strain, with lower transcription levels of *nosZ* and less N_2_O production by NO_2_^–^ reduction (Figure 1 and Supplementary Figure 6). It has been suggested in previous studies that the accumulation of N_2_O during denitrification depends on the difference in rates of its production and reduction, but pH increase only lowered its reduction rate without any significant effect on its production upon NO_2_^–^ reduction (Betlach and Tiedje, 1981; Liu et al., 2010; Han et al., 2019). In our study, the pronounced decrease in N_2_O reduction at concentrations, 0.69 to 0.93 to mmol L^–1^ and pH 9.8 can explain the observed accumulation of N_2_O (Figures 3, 5). In contrast, transient to permanent N_2_O accumulation at slightly acidic conditions (pH 6.0) has been repeatedly demonstrated for denitrifiers like *Paracoccus denitrificans* and *Shewanella loihica* (Bergaust et al., 2010; Liu et al., 2014; Kim et al., 2017). Variable patterns of reduced affinities of *nosZ* to N_2_O and reduced copy numbers of *nosZ* can explain the relevant decrease in its reduction rates. The transcription of *nosZ* was unaffected at optimal pH while it was lower at alkaline pH during denitrification in other studies (Liu et al., 2014; Kim et al., 2017; Han et al., 2019). Nitric oxide, not analyzed in our studies, has known toxicity to many microorganisms and can inhibit the activity of key enzymes including nitrous oxide. Based on the results obtained in this study, the observed decrease in the rates of denitrification by *Thauera* strains can be overall explained by inhibition of denitrification enzyme activities caused by lower or delayed transcription of genes encoding these enzymes and reduced affinity of these enzymes to substrates at tested high pHs.

### Implications of Increased pH on Industrial Applications

Other researchers have shown the importance of thermophilicity, salinity and electron donors in NO_3_^–^-mediated industrial applications (Fida et al., 2016; An et al., 2017; Chen et al., 2017). This study provides information on the impact of N sources, their initial concentrations, and pH. The observed accumulation of NO_2_^–^ and N_2_O upon NO_3_^–^ reduction at increased pH is desirable in control of sulfide formation in soured oil fields. Although the results from our experiments with NO_2_^–^ as a sole electron acceptor suggested that the use of NO_2_^–^ to inhibit SRB would be preferable to the use of NO_3_^–^, this may not be favorable in other applications, such as wastewater treatment. In such cases, this reaction can readily cause damage to other beneficial microorganisms and aquatic life through increasing NO_2_^–^ concentrations over time or discharge of NO_2_^–^ rich waters in different aquifers (Philips et al., 2002; Zhou et al., 2011). Also, in applications such as MEOR, the reduction of NO_2_^–^ to N_2_ is required for enhanced oil production (Gassara et al., 2015; Suri et al., 2019).

Based on our findings, we hypothesize that NO_3_^–^ injections require careful adjustment of pH (e.g., liming) of oil reservoir waters to an alkaline pH range that could maintain a longer presence of denitirificaiton intermediates (NO_2_^–^ and/or N_2_O) for more effective souring control even at mesophilic conditions. On the other hand, maintaining optimal pH levels to facilitate complete reduction of NO_3_^–^ to N_2_ for activating maximum dissolved and free phase gas pressure for applications like MEOR could be managed by careful selection of N-sources and/or pH buffering.

The microbial communities in these applications are often complex and consist of actively interacting and competing bacterial species. Since the knowledge obtained here is based on studies of a single genus and a limited number of model strains, the suggested roles of these parameters in practical applications needs to be tested rigorously in laboratory to obtain a further proof-of-concept. More information is needed on the regulatory controls on exhibition of denitrification outcomes by wide range of denitrifiers from different ecosystems. Our studies may not be able to account for variations in denitrification outcomes as a response to changed physiological conditions in these scenarios. However, results of this work provide a solid basis for future research and long-term evaluations of correlations between N-source, alkalinity, pH, and end products of denitrification in various industrial systems.

## Data Availability Statement

The datasets presented in this study can be found in online repositories. The names of the repository/repositories and accession number(s) can be found in the article/Supplementary Material.

## Author Contributions

NS planned and conducted the experiments, collected, analyzed, and interpreted the data, drafted and revised the manuscript. YZ helped in conducting experiments, analyzing data and revising the manuscript. LMG supervised the work through ideas and discussions and revised the manuscript. MCR supervised the work, discussed obtained data, revised and approved the manuscript to be published. All authors contributed to the article and approved the submitted version.

## Conflict of Interest

The authors declare that the research was conducted in the absence of any commercial or financial relationships that could be construed as a potential conflict of interest.

## Footnotes

1 https://www.dsmz.de

2 https://www.snapgene.com/

3 https://www.ncbi.nlm.nih.gov/
