# Supplementary material for: Denitrification Biokinetics: Towards Optimization for Industrial Applications
Source: Front Microbiol. 2021 May 5;12:610389. doi: 10.3389/fmicb.2021.610389 (PMC8131540; doi:10.3389/fmicb.2021.610389)
Supplement: Supplementary file 1 [file Data_Sheet_1.PDF]

Supplementary Material

Denitrification Biokinetics: Towards Optimization for Industrial Applications

Navreet Suri[1*], Yuan (Mary) Zhang[1], Lisa M. Gieg[2] and M. Cathryn Ryan[1]
[1]Department of Geosciences, University of Calgary, Calgary, Alberta, Canada
[2]Department of Biological Sciences, University of Calgary, Alberta, Canada

S1 Supplementary Figures

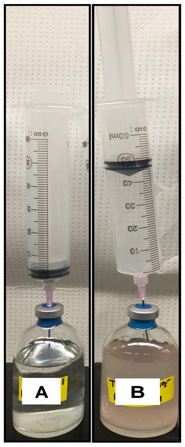

Supplementary Figure S1 | Equilibration of gas pressure (CO_2, N_2O and N_2) in the culture bottles generated through denitrification by *Thauera* strains to barometric pressure (~0.9 atm). The bottles contained 1 mmol L^{-1} each of nitrate and acetate either without (A) or with (B) inoculum.

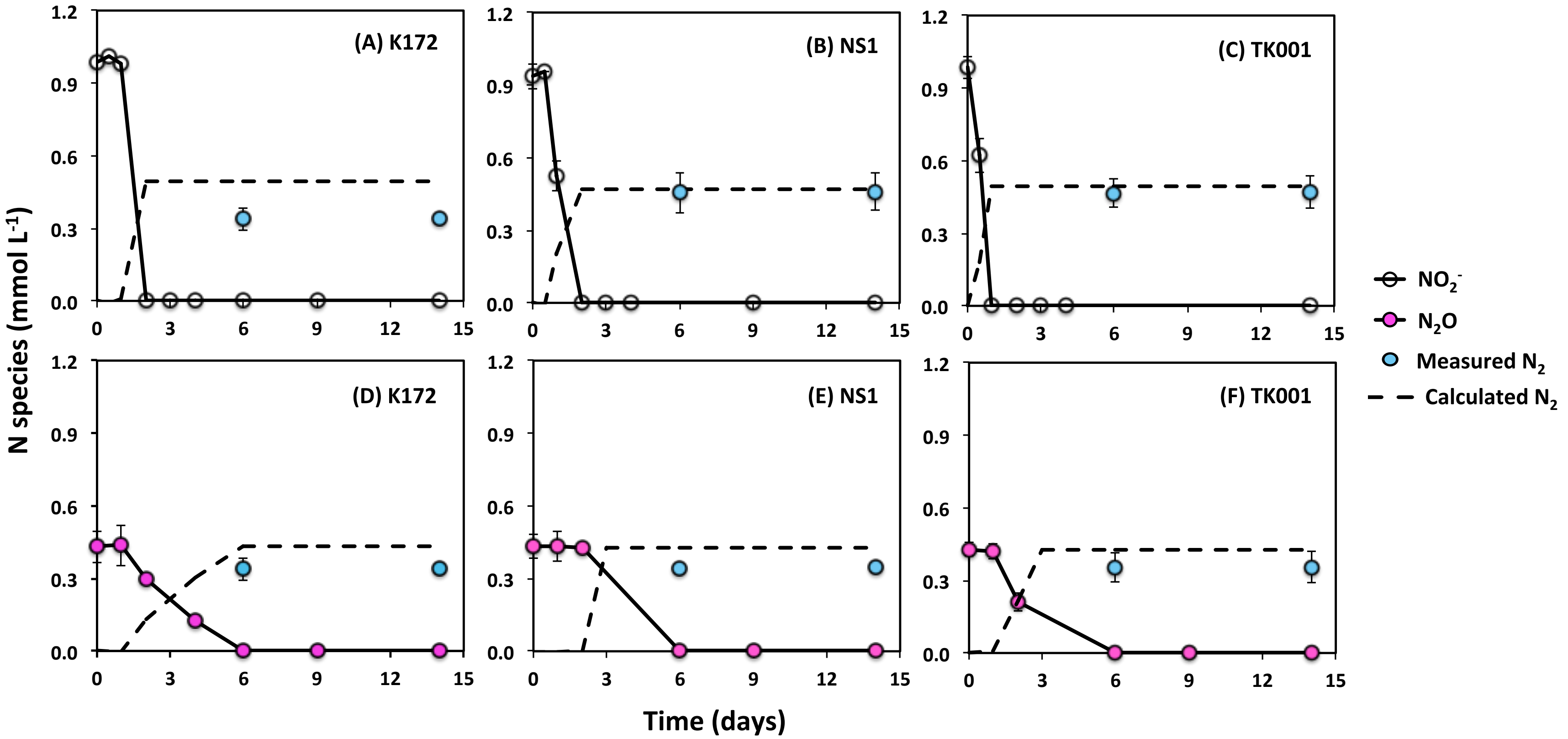

Supplementary Figure S2| Measured N species concentrations with time in batch cultures of denitrifying *Thauera* strains amended with initial concentrations of nitrite (NO_2^-), or N_2O. The bottles had a headspace of 99.9% Helium. Changes in the concentrations of NO_2^- in the aqueous phase and N_2O in the headspace were monitored during incubation at 30°C. The average N_2 produced in the headspace of these cultures was measured upon depletion of electron acceptors added initially (i.e. six days) and at the end of incubation period. Error bars represent the standard deviations at 0.05% confidence level for two to four replicates.

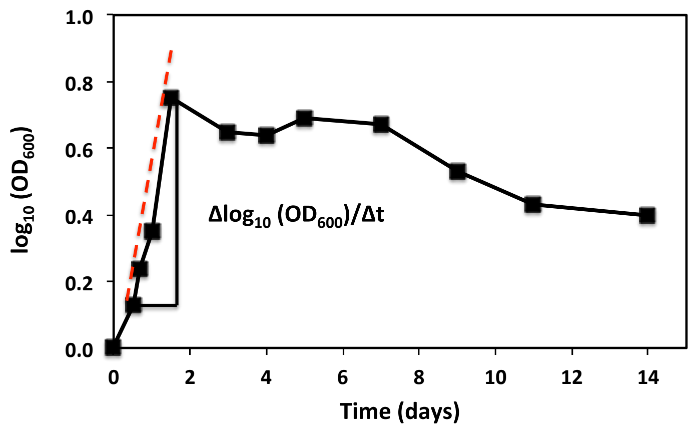

Supplementary Figure S3| Sample of the approach used to estimate the growth rates of denitrifying *Thauera* strains from the logarithmically transformed growth curves (using data collected from experimental set 1, **Table 2**).

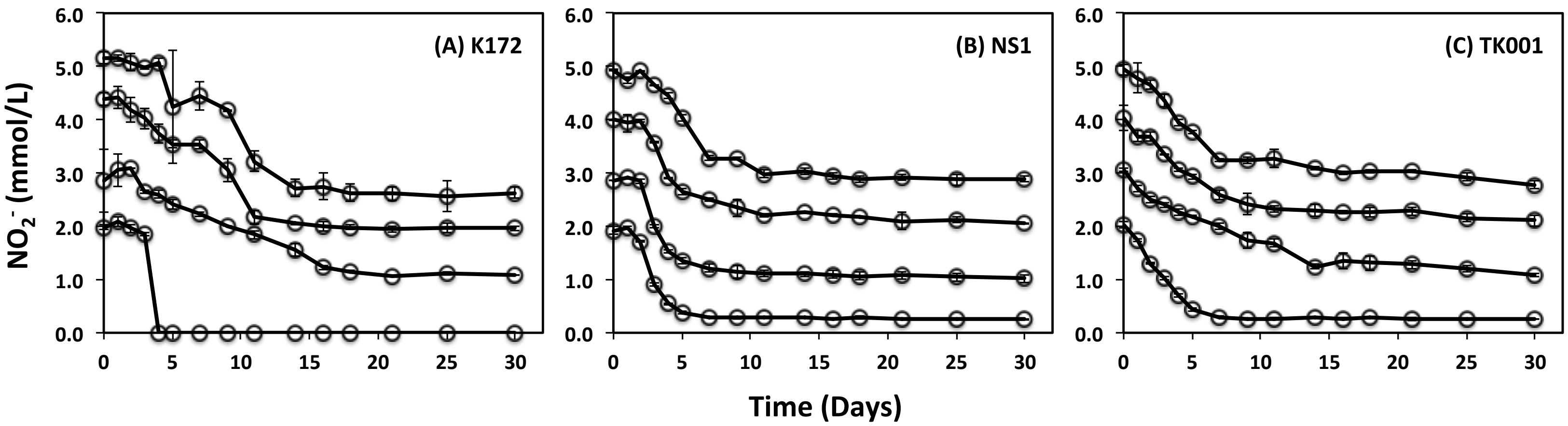

Supplementary Figure S4| Nitrite concentrations with time for 30 day incubation for varying initial NO_2^- concentrations (~ 2 to 5 mmol L^{-1}) in batch cultures of denitrifying *Thauera* strains coupled to acetate oxidation (at 30°C). Error bars represents the standard deviations at 0.05% confidence level for three to four replicates. The percent NO_2^- remaining in each of these microcosms and final concentrations of N_2O and N_2 are shown on **Figure 3**.

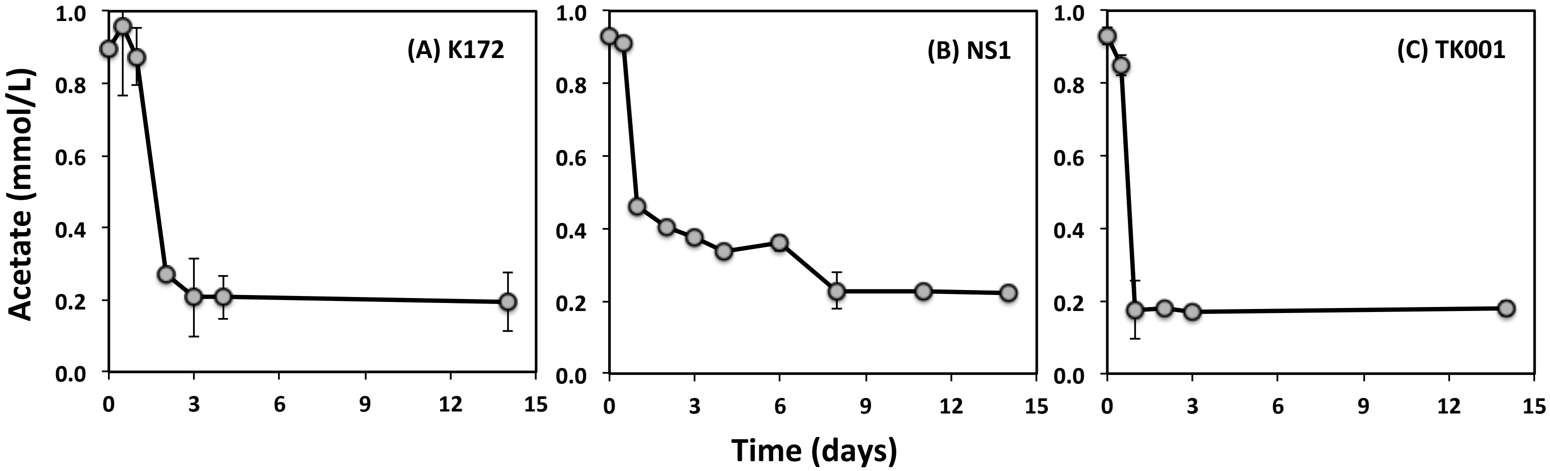

Supplementary Figure S5| Acetate oxidation in batch cultures by denitrifying *Thauera* strains coupled to reduction of nitrate to N_2 (**Figure 1**). Error bars represents the standard deviations at 0.05% confidence level for three to four replicates.

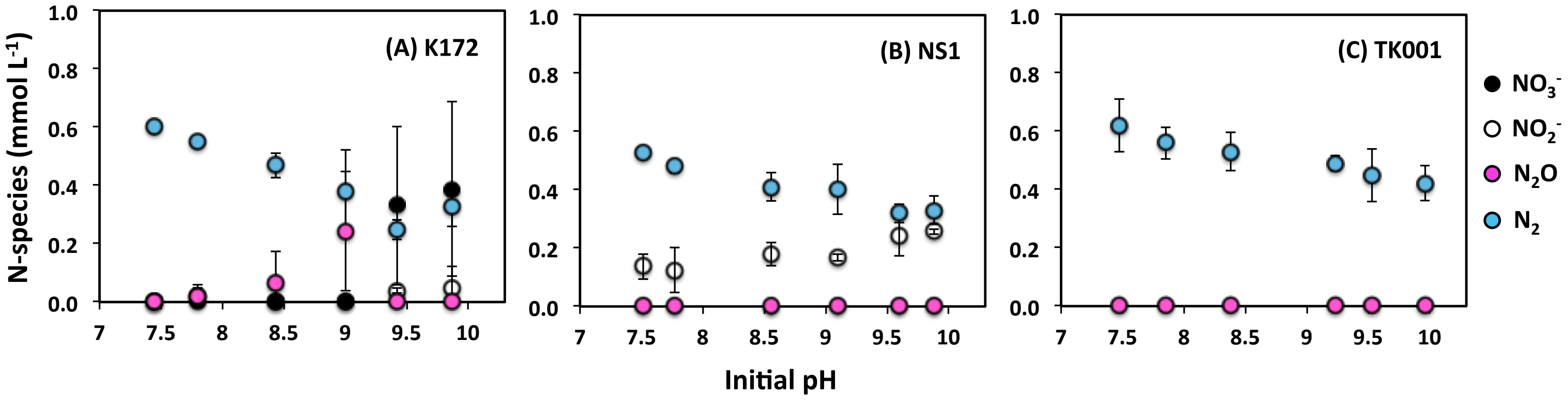

Supplementary Figure S6| Nitrate reduction in batch cultures amended with 1 mmol/L nitrate by denitrifying *Thauera* strains coupled to acetate oxidation as a function of increasing total alkalinity. Error bars represents the standard deviations at 0.05% confidence level for three replicates.

S3 Supplementary Calculations

Alkalinity is a measure of the capacity of aqueous phase in a contained system to neutralize acids. In an anaerobic closed carbonate system such as the culture bottles from our experiments (section 2.2 and 3.3), bicarbonates (HCO_3^-) and carbonates (CO_3^{2-}) contribute most to the alkalinity of a system. Assuming the contribution of other ionic species in aqueous phase of these cultures to be minimal total alkalinity was calculated in mg/L and was expressed as meq/L using equations S1 and S2.

$$[\text{Total alkalinity}] = [HCO_3^-] + 2\,[CO_3^{2-}] + (K_w/[H^+]) - [H^+] \qquad \text{(S1)}$$

$$\text{Alkalinity (meq/L as } CaCO_3) = [\text{Total alkalinity}]/50 \qquad \text{(S2)}$$

where $K_w = [H^+]\,[OH^-] = 1.47 \text{ X } 10^{-14}$ at 30°C for pure water and was not adjusted to account for the other ionic species in the aqueous phase to use in the calculations. And the HCO_3^- and CO_3^{2-} concentrations were calculated using the measured pH values (equation S3), the dissociation constants at 30°C; K1 and K2 and the equilibrium equations (equation S4 and S5) as follows.

$$pH = \log\,[H^+] \qquad \text{(S3)}$$

$$K1 = [H+]\,[HCO3-]/[CO2] = 10^{-6.33} \qquad \text{(S4)}$$

$$K2 = [H+]\,[CO32-]/[HCO3-] = 10^{-10.29} \qquad \text{(S5)}$$
